# Kinetic Description of Viral Capsid Self-Assembly Using Mesoscopic Non-Equilibrium Thermodynamics

**DOI:** 10.3390/e27030281

**Published:** 2025-03-08

**Authors:** Jason Peña, Leonardo Dagdug, David Reguera

**Affiliations:** 1 Physics Department, Universidad Autónoma Metropolitana-Iztapalapa, Mexico City 09340, Mexico; dll@xanum.uam.mx; 2 Departament de Física de la Matèria Condensada, Universitat de Barcelona, Martí i Franquès 1, 08028 Barcelona, Spain; dreguera@ub.edu; 3 Universitat de Barcelona Institute of Complex Systems (UBICS), Martí i Franquès 1, 08028 Barcelona, Spain

**Keywords:** non-equilibrium thermodynamics, entropy production, kinetic equation, Fokker–Planck equation, self-assembly, viral capsid, nucleation, nucleation rate, physical virology

## Abstract

The self-assembly mechanisms of various complex biological structures, including viral capsids and carboxysomes, have been theoretically studied through numerous kinetic models. However, most of these models focus on the equilibrium aspects of a simplified kinetic description in terms of a single reaction coordinate, typically the number of proteins in a growing aggregate, which is often insufficient to describe the size and shape of the resulting structure. In this article, we use mesoscopic non-equilibrium thermodynamics (MNET) to derive the equations governing the non-equilibrium kinetics of viral capsid formation. The resulting kinetic equation is a Fokker–Planck equation, which considers viral capsid self-assembly as a diffusive process in the space of the relevant reaction coordinates. We discuss in detail the case of the self-assembly of a spherical (icosahedral) capsid with a fixed radius, which corresponds to a single degree of freedom, and indicate how to extend this approach to the self-assembly of spherical capsids that exhibit radial fluctuations, as well as to tubular structures and systems with higher degrees of freedom. Finally, we indicate how these equations can be solved in terms of the equivalent Langevin equations and be used to determine the rate of formation and size distribution of closed capsids, opening the door to the better understanding and control of the self- assembly process.

## 1. Introduction

Viruses have become widespread throughout the world. These tiny cellular-dependent agents are found to be integral components of essentially all ecosystems on the Earth [1,2]. They are believed to invade all life forms in the sea [3]; to assist microbes that contribute to global nutrient cycles [4,5]; and to exist in highly hostile environments [6,7], including terrestrial hot springs [8], alkaline lakes [9], and the hyper-saline Dead Sea [10]. These findings have led to the consideration of viruses as the most prolific biological entities [11,12,13], attracting the attention of researchers from various scientific fields to investigate their origins, evolution, and physicochemical properties.

In particular, for almost a century or beyond, viruses and virus-like particles have intrigued physicists due to their striking structures and physical properties [14,15,16,17,18], mechanisms of self-assembly [19,20,21,22], and strategies for the packaging and delivery of nucleic acids [23,24,25,26], among other aspects. Similarly, keen interest has arisen in the self-assembly of other spherical and tubular biological and synthetic shells [27,28,29,30,31], such as carboxysomes [32,33]. However, despite the increasing interest in the self-assembly of viruses and the numerous remarkable and insightful studies on this topic (see, for example, refs. [34,35,36,37,38,39,40,41,42,43,44,45,46,47,48,49,50,51,52,53,54,55,56,57,58,59]), many of the underlying physical mechanisms involved in the viral replication cycle remain poorly understood.

In essence, viruses are composed of a single or multiple chains of nucleic acids wrapped in a protein shell, also known as the capsid [60,61]. Their genomes encode a limited set of instructions that induce the synthesis of one or a few types of capsid proteins (CPs), which constitute the capsid. Additionally, viral genomes compel the host cell to produce copies of the virus’s nucleic acids, which will be enclosed within the capsid, thereby enabling the new viral particles to hijack other susceptible cells [60].

The economy and efficiency of viral capsid self-assembly (VCSA) are fascinating and exceptional. Under appropriate conditions, a collection of CPs can aggregate to form a capsid with a structure identical to or very similar to that of native viruses, even in vitro and in the absence of genetic material [62,63,64,65,66,67,68,69,70]. Furthermore, numerous experimental studies show that viral capsids and other nanostructures exhibit polymorphism [50,62,66,71,72,73,74,75], which means that, under certain conditions, the resulting assembled structures manifest differences in size, shape, and symmetry. Polymorphism can sometimes be inferred from the system’s energy surface, which may reveal multiple minima indicating the potential observation of different structural forms [76]. Scientists have successfully reproduced key features of this phenomenon using theoretical and computational approaches [50,76,77,78,79,80,81,82,83,84,85,86]. These include the formation of icosahedral capsids and other geometrical shapes [80], the necessity of well-defined metastable intermediates for self-assembly [82], the distribution of malformed capsids [85], and even control over the shapes of capsids using DNA origami nanostructures as binding and assembly platforms [86]. Nonetheless, there are limitations in the current understanding of the kinetic pathways involved in this process, their role in polymorphism, and the ongoing debate on whether viral capsid self-assembly and polymorphism, in some viruses, are driven by kinetics or thermodynamics [36,38,50,66,87]. More importantly, despite the usefulness of certain kinetic models, often based on the proposal and numerical solution of a large set of reaction equations, these models have significant drawbacks that severely limit their overall predictive capacity [88]. Such limitations may bias or underestimate the role of kinetics in the self-assembly process, as well as its influence on the size distribution of native-like assemblies and aberrant structures or their relative populations observed experimentally [89].

Classical nucleation theory of virus capsids (CNTVC) [87] stands out as an alternative approach that, by relying on the physical modeling of the free energy of capsid formation, overcomes some of these limitations [88] and provides a robust framework for both assembly and disassembly modeling [90]. In the standard description of viral assembly provided by CNTVC, the radius of a growing capsid is fixed and only the number of CPs, or more generally of capsid building blocks (CBBs), which can be clusters of CPs, is used as a single relevant reaction coordinate. However, the radius and aspect ratio of a growing shell are fluctuating variables that might lead to the formation of a final closed structure that is very different from the energetically optimal assembly. In addition, viral self-assembly is fundamentally an out-of-equilibrium process. Hence, the kinetic aspects are very important and require the formulation of proper equations to describe them. This highlights the necessity of a formalism that facilitates the study of the non-equilibrium kinetics of viral capsid formation.

In this context, mesoscopic non-equilibrium thermodynamics (MNET) stands out as the ideal framework to rigorously formulate kinetic equations to describe out-of-equilibrium processes at the nanoscale, where fluctuations are unavoidable.

In this article, we combine CNTVC and MNET to derive the equations governing the non-equilibrium kinetics of viral capsid formation. For this purpose, we first review some of the main features of VCSA, suggesting that nucleation is the underlying mechanism driving this phenomenon. Then, we summarize the fundamental ideas behind CNTVC and briefly discuss the resulting free energy landscape for the formation of spherical (icosahedral) and tubular (elongated) capsids. Thereafter, we provide a comprehensive overview of the general framework of MNET, where entropy production is key to describe the time evolution of an out-of-equilibrium system. Subsequently, we derive a general Fokker–Planck equation describing the kinetics of viral self-assembly with arbitrary degrees of freedom. We then describe in detail the case of the self-assembly of a spherical capsid with a fixed radius, which corresponds to a single degree of freedom, and indicate how to extend this approach to capsids with different architectures and energetic contributions and higher degrees of freedom. Following this, we explore the determination of the size distribution of closed capsids and the computation of steady-state nucleation rates as further applications of this alternative theoretical framework. Finally, we provide our conclusions and a future outlook on the application of MNET to describe VCSA.

## 2. Kinetic Aspects of Viral Capsid Self-Assembly

In the context of viral capsid formation, the study of the kinetics aims to describe how and how quickly closed capsids self-assemble. To develop a robust kinetic theory that successfully reproduces, at least qualitatively, the general aspects of VCSA, it is necessary to understand the actual behavior of this process. Numerous studies on capsid formation reveal two fundamental characteristics of its kinetics. Initially, there is a delay period during which no capsids are produced, even under conditions that promote self-assembly. Subsequently, the production of capsids increases rapidly, following a sigmoidal curve [77,87,91,92]. The disassembly of the capsid occurs under different physicochemical conditions than those for assembly, leading to a hysteresis phenomenon [93,94]. In other words, once capsids are formed and closed, they do not dissolve easily, making viral capsids resistant to hazardous conditions. Based on these characteristics, several kinetic models have been proposed to describe the self-assembly of viral capsids. They have been used to describe the assembly of viruses such as hepatitis B [66] and C [95], human papillomavirus (HPV) [73], and brome mosaic virus (BMV) [96], as well as to study the influence of nucleic acids and lipid bilayers on the assembly [97]. These contributions have shed light on the underlying mechanisms used by CBBs to assemble complex structures.

The motivation to develop an alternative theoretical framework for the kinetics of VCSA arises from the strong (and somewhat artificial) approximations and assumptions used in some kinetic models. For example, modeling the kinetics of viral capsids with a large set of master equations requires significant computational resources and accurate knowledge of all attachment and detachment rates of CBBs as input [88], but these are typically unknown and must therefore be inferred. Additionally, this approach relies on experimental data and highly specific input parameters for each modeled capsid. This complexity makes the theoretical framework difficult to generalize to situations where the radii of spherical and tubular capsids are not fixed [98], as well as to other macromolecules exhibiting self-assembly.

The kinetic theory that arises from the continuum description of VCSA given by CNTVC stands out as an alternative approach. By relying on the physical modeling of the free energy of capsid formation, this theory is able to overcome some of these limitations and provide closed solutions for (i) the critical size of the capsid, (ii) the nucleation barrier, and (iii) the steady-state nucleation rate [87]. Nonetheless, the standard kinetic description of CNTVC focuses on the formation of capsids with a fixed geometry and radius, using a single variable—typically, the number of CBBs in the growing capsid—as a reaction coordinate. This description may be valid as a first approximation, but, during the assembly of a virus, there might be fluctuations in the radius or shape of the growing shell, which can impact dramatically the final outcome of the process. The generalization of a master equation approach to incorporate these fluctuations involves a substantial amount of mathematics, and it becomes increasingly complex when modeling VCSA with multiple degrees of freedom or two or more types of CBBs [98].

In light of these limitations, MNET can provide kinetic equations to model out-of-equilibrium processes, thereby establishing a systematic approach to analyzing the non-equilibrium properties of the system based on the knowledge of its behavior at equilibrium. This aspect positions MNET as a promising theoretical framework for the description of the out-of-equilibrium characteristics of the self-assembly of not only tubular and spherical viral capsids but also carboxysomes and synthetically assembled structures.

Mesoscopic non-equilibrium thermodynamics relies on knowledge of the equilibrium free energy landscape of the out-of-equilibrium process that one aims to describe. Therefore, the energetic considerations involved in the context of viral capsid formation are discussed briefly in the next section.

## 3. Continuum Description of the Free Energy Landscape of Viral Capsid Self-Assembly

**Figure 1:**
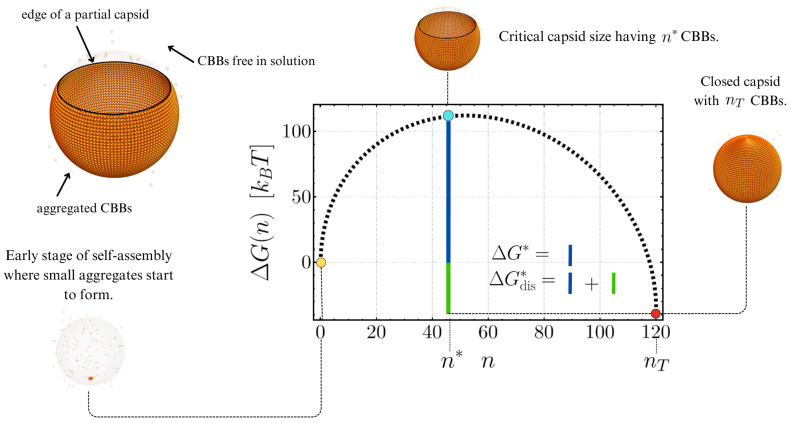
Schematic representation of a partial capsid having *n* CBBs (top left corner) and the free energy landscape generated by ΔG(n) in Equation (2) as a function of the number of CBBs, *n*. The self-assembly of viral capsids is described from left to right, while the disassembly of closed capsids occurs from right to left. The nucleation barrier towards assembly, $\Delta G^{*}$, differs from the nucleation barrier driving the disassembly of closed capsids, $\Delta G_{\mathrm{dis}}^{*}$, reflecting the system’s hysteresis.

The primary hypothesis of the continuum description of VCSA is that nucleation is the underlying mechanism for the aggregation of CBBs into closed protein shells [87]. Nucleation is an activated process that considers self-assembly as the overcoming of a free energy barrier to capsid formation. In the original formulation of CNTVC, the self-assembly of a spherical capsid is modeled as the growth of a partial shell that can increase or decrease in area due to the attachment or detachment of individual CBBs. An intermediate aggregate is represented as a spherical cap with fixed radius $R_{T}$ equal to the final radius of the closed capsid. The growth of this spherical shell is promoted by the energy gain Δμ due to the aggregation of CBBs and hindered by an energy penalty due to the missing contacts at the edge of the growing shell (see Figure 1). The resulting free energy of the self-assembly of empty spherical capsids, ΔG, is given by [87]

(1) ΔG=nΔμ+τl(n),

where *n* is the instantaneous number of CBBs in a partial capsid, τ is the line tension (analogous to the surface tension of a liquid droplet in homogeneous condensation), and l(n) is the length of the rim. Taking into account the geometry of a spherical cap, the previous expression can be rewritten as [45,87]

(2) $$\Delta G\left(n\right)=n\Delta \mu +\lambda \sqrt{n(n_{T}-n)},$$

in which $n_{T}$ is the total number of CBBs in the closed capsid, and $\lambda =4\pi \tau R_{T}/n_{T}$ is an edge energy constant related to the line tension, τ. The competition between these opposite contributions leads to the existence of a nucleation barrier, which has to be surmounted in order to assemble a fully formed capsid.

In Figure 1, we show a schematic representation of a partial capsid having *n* CBBs (top left corner) and a representative energy landscape for $\Delta \mu =-0.5\;k_{B}T$ and $\lambda \approx 2.15\;k_{B}T$, where three important stages of VCSA are highlighted. According to Equation (2), capsid formation starts at (n=0,ΔG=0) (yellow point), representing the situation where all CBBs are free in the solution. As the CBBs start to aggregate due to the thermal fluctuations of the bath, the free energy of the incipient shell increases, indicating that partial capsids with a small number of constituents are unstable assemblies that will tend to dissolve. Only when having a cluster with a critical size $n^{*}$ does the system reach an unstable state (cyan point) where any perturbation can cause the partial capsid to dissolve or continue assembling until it closes. If the nucleation barrier, $\Delta G^{*}$, is surmounted, the aggregate will tend to progressively grow, ultimately forming a closed spherical capsid of size $n_{T}$ (red point).

This continuum description of VCSA qualitatively aligns with theoretical, experimental, and computational findings regarding VCSA [87], including the existence of a critical capsid size, the lack of a significant population of intermediate capsids, and the presence of hysteresis in the system. The latter inference arises from the fact that, for Δμ<0, $\Delta G^{*}\neq \Delta G_{\mathrm{dis}}^{*}$, with $\Delta G_{\mathrm{dis}}^{*}$ being the energetic barrier to be surmounted for disassembly, as shown in Figure 1.

### Extensions of Classical Nucleation Theory of Viral Capsids Incorporating Elastic Contributions

The standard classical nucleation theory (CNT) description of viral assembly, as shown in Equation (2), only considers the binding energy gain and line tension costs involved in the formation of spherical capsids with a predetermined radius. This picture neglects an important ingredient: the elastic energy arising from the bending and stretching associated with the growth of a curved shell. Incorporating these contributions leads to the following free energy of formation [99,100,101]:

(3) $$\Delta G=n\Delta \mu +\tau \;l\left(n\right)+G_{\mathrm{ela}},$$

where $G_{\mathrm{ela}}=G_{\mathrm{str}}+G_{\mathrm{bend}}$ is the elastic energy associated with both stretching, $G_{\mathrm{str}}$, and bending, $G_{\mathrm{bend}}$, energies introduced by the curvature of the capsid. The mathematical expressions for the stretching and bending contributions within $G_{\mathrm{ela}}$ depend on the specific geometry of the growing aggregate but are generally modeled using thin shell theory (TST) [102,103,104] and the Helfrich model [105], respectively.

Several studies have explored the elastic properties of viral capsids and complex macromolecules along this line [42,102,103,106,107,108,109,110,111,112,113]. The incorporation of these terms not only modifies the values of the nucleation barrier to surmount but also might alter the radius of the shell and even the preferred geometry of the growing capsid. The competition between the line tension, the difference in chemical potential, and the relative importance of bending and stretching contributions, quantified by the Föppl–von Kármán (FvK) number, $\gamma_{\mathrm{FvK}}$, can result in the formation of spherical caps but also ribbons and cylindrical patches [99].

At the limit where bending dominates and stretching can be neglected, the free energy of formation of spherical capsids becomes [100,101]

(4) $$\Delta G\left(n,R\right)=\lambda \;n_{T}\left\{n_{r}\left[f_{r}{\left(\frac{1}{R_{r}}-1\right)}^{2}-\Gamma \right]+\sqrt{n_{r}\left(1-\frac{n_{r}}{R_{r}^{2}}\right)}\right\}.$$

The derivation of the latter equation is described in detail in Ref. [101] and involves the use of scaled variables and parameters. Specifically, in this formulation, $n_{r}\equiv n/n_{T}$ and $R_{r}\equiv R/R_{T}$, where *R* is the instantaneous radius of the capsid, whereas Γ≡−Δμ/λ and $f_{r}\equiv 2\pi \kappa /\lambda n_{T}$ are the scaled supersaturation and the relative bending constant, respectively. The new parameters introduced in this extension of CNTVC include κ, which is the bending modulus of the capsid, and $n_{T}$ and $R_{T}$, representing the target number of CBBs and the preferred curvature radius of a capsid, both dictated by the preferred angle of interaction between individual CBBs [101]. In the latter equation, ΔG, depends on both *n* and *R* as the model allows changes in the curvature radius of a partial capsid as it progressively grows.

At the limit dominated by stretching energy, i.e., $\gamma_{\mathrm{FvK}}>>1$, only tubes are formed as these structures can freely grow without any stretching energy penalty. If the line tension is also large, the cylindrical tubes will form from curved square or circular patches, which are the shells minimizing the contour length. In this case, the free energy of formation is found to be [99,114]

(5) $$\Delta G\left(n,R\right)=n\Delta \mu +\tau \;l\left(A\right)+\frac{1}{2}\frac{\kappa A}{R_{T}^{2}}\left[{\left(\frac{1}{R_{r}}-1\right)}^{2}+1\right],$$

where $A=na_{1}$ represents the area of the patch composed of *n* CBBs, each with nominal area $a_{1}$. This free energy landscape for tube formation also depends on two relevant variables: the radius of the growing tube and the number of CBBs. However, if the line tension is small, these tubes will form, in general, from rectangular (or elliptical) curved patches described by three variables: the number of CBBs, *n*; the radius of the growing tube, *R*; and the aspect ratio of the patch, *f*. In terms of these parameters, the free energy of formation of a tube from of a rectangular patch can be written as [114]

(6) $$\Delta G_{\mathrm{rect}}\left(n,R,f\right)=n\Delta \mu +\Lambda \frac{\left(1+f\right)}{\sqrt{f}}\sqrt{n}+\frac{1}{2}\tilde{\kappa }n\left[{\left(\frac{1}{R_{r}}-1\right)}^{2}+1\right],$$

where $\Lambda \equiv 2\tau \sqrt{a_{1}}$ is a line energy constant and $\tilde{\kappa }\equiv \kappa a_{1}/R_{T}^{2}$ is a bending stiffness constant, which are redefined for convenience. The last term in Equation (6) represents the bending energy cost and has been obtained using the continuum elasticity model introduced by Castelnovo [113], which is a generalization of Helfrich’s model [105] for systems with non-zero spontaneous curvature.

All free energies presented above represent extensions of CNTVC that explore different aspects of the self-assembly of spherical (icosahedral) and cylindrical (tubular) assemblies. More specifically, they provide valuable insights for the study of the equilibrium aspects of the self-assembly process. In particular, at equilibrium, the probability density of finding a partial shell with a given size is given by the standard statistical mechanics expression [88,98,115]

(7) $$P_{\mathrm{eq}}∼\exp\left(\frac{-\Delta G}{k_{B}T}\right),$$

which, naturally, is a Boltzmann factor. This equation is a key component that will allow us to derive the entropy production of the system and, subsequently, the equations describing the non-equilibrium kinetics of VCSA.

The presence of additional degrees of freedom in the free energy landscape (as described in Equations (4)–(6)) has important consequences for the energetics of capsid formation and the definition of the optimal size and radius of the resulting spherical or tubular structure. These energetic considerations have been analyzed in Refs. [100,101,114]. However, since self-assembly is a dynamic process, these additional degrees of freedom do not only impact the energetics of the capsid. The rate at which a shell changes its radius or aspect ratio can cause the pathway of assembly to deviate from the optimal one (i.e., the minimum free energy path), yielding a distribution of sizes that may be quite different from the expected optimal aggregate. Thus, it is very important to describe the multidimensional kinetics of viral assembly when other degrees of freedom become relevant. This is accomplished using MNET.

In the next section, we briefly review the general framework of MNET [116]. Then, we particularize it to describe the behavior and evolution of the out-of-equilibrium mechanisms driving VCSA.

## 4. General Framework for Mesoscopic Non-Equilibrium Thermodynamics

### Entropy as the Key to the Time Evolution of an Out-of-Equilibrium System

MNET is a theoretical framework that extends non-equilibrium thermodynamics to systems that exist between the microscopic and macroscopic scales, where fluctuations are significant and non-linear effects dominate [116]. Unlike traditional approaches, which are limited to macroscopic systems and linear responses, MNET incorporates a probabilistic interpretation of densities, conservation laws, and entropy principles to describe a broader range of non-equilibrium phenomena. In this context, MNET extends traditional thermodynamic concepts to systematically analyze the stochastic behavior of small systems. Readers interested in the foundations of MNET are referred to [116].

In a nutshell, the main asset of MNET lies in acknowledging that entropy production is key to describing the evolution of a system out of equilibrium. To truly understand this core component and its implications in kinetics, consider a system with *i* degrees of freedom, $\{\gamma_{i}\}=\left\{\gamma_{1},\gamma_{2},\cdots ,\gamma_{i}\right\}$, representing any relevant set of coordinates or mesoscopic degrees of freedom that define the state of the system. For example, i=1 and $\gamma_{1}=n$ for the standard one-dimensional description of CNTVC (see Equation (2)) or i=2 and $\{\gamma_{i}\}=\left\{n,R\right\}$ if we are characterizing the self-assembly kinetics of a protein shell whose number of constituents and curvature radius vary with thermal fluctuations (as in Equation (4) or Equation (5)). Given that we are examining a statistical system, we must work with probabilities. The probability density in terms of finding the system in the state {γ,γ+δγ} at time *t* is P(γ,t). The entropy of the system in terms of this probability is expressed by the Gibbs entropy postulate, namely [117]

(8) $$S=S_{eq}-k_{B}\int P\left(\gamma ,t\right)\ln\left[\frac{P(\gamma ,t)}{P_{\mathrm{eq}(\gamma )}}\right]d\gamma ,$$

where $S_{\mathrm{eq}}$ is the entropy of the system when the degrees of freedom {γ} are at equilibrium. The second term of the latter equation considers entropy contributions that arise when the {γ} variables are out of equilibrium, which, as a consequence, produce a deviation in the probability density P(γ,t) from its equilibrium value, $P_{\mathrm{eq}}(\gamma )$. The equilibrium probability density is typically constructed to fulfill the following condition [118]:

(9) $$P_{\mathrm{eq}}∼\exp\left[\frac{-\Delta W(\gamma )}{k_{B}T}\right].$$

This is a relation in which ΔW(γ) is the reversible work or availability required to achieve the particular state defined by γ; $k_{B}$ is Boltzmann’s constant; and *T* is the temperature.

Variations in Equation (8) lead to

(10) $$\Delta S=-k_{B}\int \Delta P\left(\gamma ,t\right)\ln\left[\frac{P(\gamma ,t)}{P_{\mathrm{eq}}(\gamma )}\right]d\gamma .$$

Since the probability is a conserved normalized quantity, the probability density in the γ−space is governed by the continuity equation, allowing us to write

(11) $$\frac{\partial P(\gamma ,t)}{\partial t}=\frac{-\partial J(\gamma ,t)}{\partial \gamma },$$

with J(γ,t) being the density flux, a function that will be specified later. By taking the time derivative of Equation (10) and using Equation (11), we obtain

(12) $$\frac{dS}{dt}=k_{B}{\left\{\ln\left[\frac{P(\gamma ,t)}{P_{\mathrm{eq}}(\gamma )}\right]J\left(\gamma ,t\right)\right\}}_{\mathrm{eval}}-k_{B}\int J\left(\gamma ,t\right)\frac{\partial }{\partial \gamma }\left\{\ln[\frac{P(\gamma ,t)}{P_{\mathrm{eq}(\gamma )}}]\right\}d\gamma ,$$

after integrating by parts. An alternative representation of the latter equation reads

(13) $$\frac{dS}{dt}=-\int \frac{\partial }{\partial \gamma }J_{s}\;d\gamma +\sigma ,$$

where we have defined the entropy flux and entropy production as

(14) $$J_{s}\equiv k_{B}\;J\left(\gamma ,t\right)\ln\left[\frac{P(\gamma ,t)}{P_{\mathrm{eq}}(\gamma )}\right],$$

and

(15) $$\sigma \equiv -k_{B}\int J\left(\gamma ,t\right)\frac{\partial }{\partial \gamma }\left\{\ln[\frac{P(\gamma ,t)}{P_{\mathrm{eq}}(\gamma )}]\right\}d\gamma ,$$

respectively. As in standard non-equilibrium thermodynamics (NET) [117,119], the entropy production is the product of a flux multiplied by a thermodynamic force. In this case, the thermodynamic force, in terms of the mesoscopic variables, is identified as the gradient of the factor $\ln[P\left(\gamma ,t\right)/P_{\mathrm{eq}(\gamma )}]$. Following the tenets of NET, we assume a linear dependence between fluxes and forces, which leads us to

(16) $$J\left(\gamma ,t\right)=-k_{B}T\;L\left(\gamma ,P\left(\gamma \right)\right)\frac{\partial }{\partial \gamma }\left\{\ln[\frac{P(\gamma ,t)}{P(\gamma )}]\right\},$$

in which L(γ,P(γ)) is an Osanger coefficient that may depend, in general, on the probability density of finding the system at a certain state γ, P(γ).

By substituting Equation (16) into Equation (11) and defining a generalized diffusion coefficient $D\equiv [k_{B}L\left(\gamma ,P\left(\gamma \right)\right)]/P\left(\gamma ,t\right)$, we find that

(17) $$\begin{array}{cc} \frac{\partial P(\gamma ,t)}{\partial t} & =-\frac{\partial }{\partial \gamma }\left\{-k_{B}\;L\left(\gamma ,P\left(\gamma \right)\right)\frac{\partial }{\partial \gamma }\ln\left[\frac{P(\gamma ,t)}{P_{\mathrm{eq}}\left(\gamma \right)}\right]\right\}\\ \; & =\frac{\partial }{\partial \gamma }[D\;P_{\mathrm{eq}}\left(\gamma \right)\frac{\partial }{\partial \gamma }\frac{P(\gamma ,t)}{P_{\mathrm{eq}}\left(\gamma \right)}], \end{array}$$

which is a diffusion equation. From this relation, we can obtain the equations governing the non-equilibrium kinetics of a system from its behavior at equilibrium. Furthermore, by implementing the equilibrium statistical description of the process into the kinetic non-equilibrium mathematical framework, i.e., Equation (9) into Equation (17), we obtain

(18) $$\frac{\partial P}{\partial t}=\frac{\partial }{\partial \gamma }[D\frac{\partial }{\partial \gamma }P\left(\gamma ,t\right)+\frac{DP(\gamma ,t)}{k_{B}T}\frac{\partial }{\partial \gamma }\Delta W\left(\gamma \right)],$$

which is the Fokker–Planck equation describing the evolution of the probability density P(γ,t), i.e., a kinetic equation describing how P(γ,t) changes in time and in the γ phase space.

Under the standard conditions for assembly, consisting of a constant pressure and temperature, the proper thermodynamic potential is the Gibbs free energy, namely ΔW≡ΔG=ΔH−TΔS, where ΔH and ΔS are the changes in enthalpy and entropy, respectively. The resulting equation is a Fokker–Planck equation for a system diffusing over the free energy landscape defined by ΔG. In the context of VCSA, the landscape features an unstable or saddle point state corresponding to the nucleation barrier that must be surmounted to self-assemble a tubular or spherical virus. Implementing the proper free energy of formation for spherical (icosahedral) capsids, i.e., Equation (2) or Equation (4), or for tubular (elongated) capsids, given by Equation (5) or Equation (6), in the general kinetic equation, i.e., Equation (18), leads to specific kinetic equations that can be used to compare the outcomes and rates of formation of different capsid architectures.

Given the structure of Equation (18), MNET considers the evolution of a system in time to be a generalized diffusion process in the space of the mesoscopic variables {γ} over an energy landscape generated by the equilibrium free energy, ΔG. The MNET framework used to model out-of-equilibrium kinetics has been successfully applied to describe many non-equilibrium phenomena, including diffusion in the presence of entropic barriers [120], activated processes [121,122,123], molecular motors [124], and the translocation of stiff chains [125].

In the following section, we particularize this general framework to describe the kinetics of VCSA in spherical and tubular viruses.

## 5. Kinetics of Viral Capsid Self-Assembly from MNET

Once the theoretical framework is outlined, the application to the description of the non-equilibrium aspects of the self-assembly of spherical and tubular shells is straightforward. As an illustrative example, in this section, we discuss the case of the self-assembly of empty spherical capsids with one significant mesoscopic variable, whose free energy is given by Equation (2). Additionally, we extend these ideas to a system with two degrees of freedom; finally, we provide guidance on how to work with more complex systems.

### 5.1. The Case of One Degree of Freedom: The Assembly of a Spherical Capsid with a Fixed Radius

In the continuum description of the self-assembly of spherical capsids of fixed radius, whose free energy of formation is given by Equation (2), there is a single mesoscopic variable: the instantaneous number of CBBs *n*. Consequently, particularizing the Fokker–Planck Equation (18) to this system, the corresponding kinetic equation is

(19) $$\frac{\partial P(n,t)}{\partial t}=\frac{\partial }{\partial n}[D_{n}\frac{\partial P(n,t)}{\partial n}+\frac{D_{n}}{k_{B}T}\frac{\partial \Delta G}{\partial n}P\left(n,t\right)],$$

where the diffusion coefficient $D_{n}$ is related to the rate of attachment of CBBs to a partial capsid, and $\Delta G=n\Delta \mu +\lambda \sqrt{n(n_{T}-n)}$. The numerical solution of Equation (19) with proper initial and boundary conditions provides valuable insights into the time evolution of the distribution of cluster sizes and the rate of formation of closed capsids for different values of Δμ and λ.

Furthermore, the Fokker–Planck equation, Equation (19), is equivalent to a Langevin equation of the form

(20) $$\frac{\partial n}{\partial t}=-\frac{D_{n}\left(n\right)}{k_{B}T}\frac{\partial \Delta G}{\partial n}+\frac{\partial D_{n}\left(n\right)}{\partial n}+\zeta_{n},$$

where $\zeta_{n}$ represents white Gaussian noise in size, satisfying $\langle \zeta_{n}\left(t\right)\rangle =0$, $\langle \zeta_{n}\left(t\right)\zeta_{n}\left(t^{\prime }\right)\rangle =2D_{n}\left(n\right)\delta \left(t-t^{\prime }\right)$, and the second term on the right-hand side represents the noise-induced drift term arising in the general situation where the diffusion coefficient $D_{n}\left(n\right)$, which is ultimately the attachment rate, depends on the size of the shell. Equation (20) can be numerically solved using a simple stochastic Euler algorithm [126]. This perspective allows VCSA to be interpreted as a diffusive process along the free energy landscape, where the system explores different physical states. In this framework, the driving force for self-assembly is given by F=−∂ΔG/∂n, while Gaussian noise represents thermal fluctuations that facilitate the overcoming of the nucleation barrier and the exploration of energetically unfavorable states. This analogy holds for each dimension in a system with *i* degrees of freedom.

Finally, the kinetic equation given by Equation (19) provides an alternative method of deriving the rate of capsid formation at a steady state. Starting from Equation (17), Equation (19) leads to a flux of the form

(21) $$J_{\mathrm{nr}}=-\left[D_{n}\;P_{\mathrm{eq}}(n)\frac{\partial }{\partial n}\frac{P(n,t)}{P_{\mathrm{eq}}(n)}\right],$$

a relation that may be rewritten as

(22) $$\frac{J_{nr}\left(n\right)}{D_{n}\;P_{\mathrm{eq}}(n)}=-\frac{\partial }{\partial n}\frac{P(n,t)}{P_{\mathrm{eq}}(n)}.$$

By integrating by parts, we find that

(23) $$\int_{n_{0}}^{n_{f}}\frac{J_{nr}\left(n\right)}{D_{n}\;P_{\mathrm{eq}}(n)}=-\frac{P(n)}{P_{\mathrm{eq}}(n)}\;{|}_{n_{0}}^{n_{f}}.$$

At the steady state, $J_{nr}$ is independent of the number of CBBs. If this is the case, the latter equation simplifies to

(24) $$J_{nr}\int_{n_{0}}^{n_{f}}\frac{dn}{D_{n}\;P_{\mathrm{eq}}(n)}=-\frac{P(n)}{P_{\mathrm{eq}}(n)}\;{|}_{n_{0}}^{n_{f}}.$$

Now, the theoretical framework of VCSA relies on the use of concentrations instead of probability distributions; to this end, it is important to note that

(25) C(n,t)=NP(n,t),

with *N* being the total number of clusters in the system, assumed to be constant. Thus,

(26) $$J_{\mathrm{nr}}\;\int_{n_{0}}^{n_{f}}\frac{dn}{D_{n}\;C_{\mathrm{eq}}(n)}=-\frac{C(n)}{C_{\mathrm{eq}}}\;{|}_{n_{0}}^{n_{f}},$$

where C(n) is the instantaneous concentration of aggregates of size *n*; $C_{\mathrm{eq}}(n)$ is the concentration of these aggregates at equilibrium; $n_{0}=0$ represents a cluster of size 0, namely free CBBs in solution; and $n_{f}=n_{T}$, where $n_{T}$ represents the aggregation number. Then,

(27) $$\begin{array}{cc} J_{\mathrm{nr}}\int_{0}^{n_{T}}\frac{dn}{D_{n}\;C_{\mathrm{eq}}(n)} & =\frac{C(n=0)}{C_{\mathrm{eq}}(n=0)}-\frac{C(n_{T})}{C_{\mathrm{eq}}(n_{T})}. \end{array}$$

The boundary conditions for the self-assembly of viral capsids are a fixed concentration of CBBs equal to the equilibrium concentration and a null concentration of fully formed capsids, which mathematically are translated into $C\left(n=0\right)/C_{\mathrm{eq}(n=0)}\approx 1$ and $C\left(n_{T}\right)/C_{\mathrm{eq}(n_{T})}\approx 0$, respectively. Thus,

(28) $$J_{\mathrm{nr}}={\left[\int_{0}^{n_{T}}\frac{dn}{D_{n}\;C_{\mathrm{eq}}(n)}\right]}^{-1}.$$

Equation (7) can be used to express the relationship between the equilibrium concentration of aggregates of size *n*, $C_{\mathrm{eq}(n)}$ in terms of the free energy of self-assembly, ΔG(n) [98,115]. The resulting equation is $C_{\mathrm{eq}}(n)=C_{s}\exp\left[-\beta \Delta G\left(n\right)\right]$, in which $C_{s}$ is the concentration of CBBs at the reference state of supersaturation. Therefore,

(29) $$J_{\mathrm{nr}}=C_{s}\left\{\int_{0}^{n_{T}}\frac{\exp[\beta \Delta G(n)]}{D_{n}}\right\}.$$

Finally, by using the steepest descent approach within the context of Becker and Döring kinetics [87,127,128], we ultimately find that

(30) $$J=C_{s}D_{n}^{*}Z\exp\left(-\beta \Delta G^{*}\right),$$

where

(31) $$Z\equiv \sqrt{\frac{W^{*}}{2\pi k_{B}T}},\;\mathrm{and}\;W^{*}=-{\left(\frac{\partial^{2}\Delta G(n)}{\partial n^{2}}\right)}_{n=n^{*}}.$$

The latter equation is identical to Equation (15) of Ref. [87]. Thus, MNET offers a direct approach to deducing the nucleation rate of an out-of-equilibrium system.

### 5.2. Two Degrees of Freedom

Let us now consider the self-assembly of an empty spherical capsid in the bending-dominated case, for which the free energy of formation is given by Equation (4). The relevant mesoscopic variables are {n,R} as the number of CBBs and the curvature radius of the spherical cap vary during capsid formation. The out-of-equilibrium kinetic equation describing this system is

(32) $$\begin{array}{cc} \frac{\partial P(n,R,t)}{\partial t}= & \frac{\partial }{\partial n}[D_{n}\frac{\partial P(n,R,t)}{\partial n}+\frac{D_{n}}{k_{B}T}\frac{\partial \Delta G}{\partial n}P\left(n,R,t\right)]\\ \; & +\frac{\partial }{\partial R}[D_{R}\frac{\partial P(n,R,t)}{\partial R}+\frac{D_{R}}{k_{B}T}\frac{\partial \Delta G}{\partial R}P\left(n,R,t\right)], \end{array}$$

where we have considered that there are no cross-effects. The latter equation is equivalent to the following set of Langevin equations:

(33) $$\frac{\partial n}{\partial t}=-\frac{D_{n}}{k_{B}T}\frac{\partial \Delta G}{\partial n}+\frac{\partial D_{n}}{\partial n}+\zeta_{n},$$

(34) $$\frac{\partial R}{\partial t}=-\frac{D_{R}}{k_{B}T}\frac{\partial \Delta G}{\partial R}+\frac{\partial D_{R}}{\partial R}+\zeta_{R},$$

where $\zeta_{n}$ and $\zeta_{R}$ represent white Gaussian noise in size and radius, respectively, satisfying $\langle \zeta_{i}\left(t\right)\rangle =0$, $\langle \zeta_{i}\left(t\right)\zeta_{j}\left(t^{\prime }\right)\rangle =2D_{i}\delta_{ij}\delta \left(t-t^{\prime }\right)$. The second term on the right-hand side of the latter equations represents the noise-induced drift term considering the general situation where the diffusion coefficients $D_{n}$ and $D_{R}$ may depend on both the size and the radius of the growing shell. As in the one-dimensional case, $D_{n}$ and $D_{R}$ are related to the rate of subunit attachment and the rate of radial fluctuation, respectively. The kinetic equation describing the formation of cylindrical assemblies from a squared patch, or any system with two mesoscopic degrees of freedom, is identical to Equation (32) and leads to the same set of Langevin equations that can be solved with a simple stochastic Euler algorithm. The appropriate free energy of self-assembly for tubular assemblies in the limit of high line tension is given by Equation (5).

### 5.3. Three Degrees of Freedom: The Assembly of Tubes with Arbitrary Line Tension

For the general case of an arbitrary value of the line tension, the aspect ratio *f* of the growing tubular shell also becomes a fluctuating variable. In this case, the free energy landscape of formation of a cylindrical assembly is given by Equation (6), and the corresponding equation that describes the kinetics of the assembly becomes

(35) $$\begin{array}{cc} \frac{\partial P(n,R,f,t)}{\partial t}= & \frac{\partial }{\partial n}[D_{n}\frac{\partial P(n,R,f,t)}{\partial n}+\frac{D_{n}}{k_{B}T}\frac{\partial \Delta G}{\partial n}P\left(n,R,f,t\right)]\\ \; & +\frac{\partial }{\partial R}[D_{R}\frac{\partial P(n,R,f,t)}{\partial R}+\frac{D_{R}}{k_{B}T}\frac{\partial \Delta G}{\partial R}P\left(n,R,f,t\right)]\\ \; & +\frac{\partial }{\partial f}[D_{f}\frac{\partial P(n,R,f,t)}{\partial f}+\frac{D_{f}}{k_{B}T}\frac{\partial \Delta G}{\partial f}P\left(n,R,f,t\right)], \end{array}$$

which is equivalent to the following set of Langevin equations:

(36) $$\frac{\partial n}{\partial t}=-\frac{D_{n}}{k_{B}T}\frac{\partial \Delta G}{\partial n}+\frac{\partial D_{n}}{\partial n}+\zeta_{n},$$

(37) $$\frac{\partial R}{\partial t}=-\frac{D_{R}}{k_{B}T}\frac{\partial \Delta G}{\partial R}+\frac{\partial D_{R}}{\partial R}+\zeta_{R},$$

(38) $$\frac{\partial f}{\partial t}=-\frac{D_{f}}{k_{B}T}\frac{\partial \Delta G}{\partial f}+\frac{\partial D_{f}}{\partial f}+\zeta_{f},$$

where $\zeta_{n}$, $\zeta_{R}$, and $\zeta_{f}$ represent white Gaussian noise in size, radius, and aspect ratio, respectively, satisfying $\langle \zeta_{i}\left(t\right)\rangle =0$, $\langle \zeta_{i}\left(t\right)\zeta_{j}\left(t^{\prime }\right)\rangle =2D_{i}\delta_{ij}\delta \left(t-t^{\prime }\right)$.

In all of these cases, the numerical solution of either the Fokker–Planck equation or the equivalent set of Langevin equations provides the probability density of finding the system in a specific state at time *t*. When evaluated at an appropriate time, it reveals the size distribution of intermediate aggregates and fully formed capsids. This facilitates the tuning of experimental and computational parameters, such as the concentration of CBBs and the binding energy between them, to explore the possibility of achieving a very efficient assembly of structures of the desired size or to induce the formation of incorrect sizes as a potential strategy to hamper viral replication. These solutions also provide insights into the preferred self-assembly pathways, the stability of intermediate aggregates, and the polydispersity and distribution of capsid sizes formed under given physicochemical conditions. Additionally, the out-of-equilibrium kinetic equation enables the direct determination of the transient and steady-state nucleation rates. These features highlight the advantages, potential applications, and universality of the MNET approach in the context of the self-assembly of viral capsids and other biological structures.

## 6. Conclusions

We have seen how an appropriate evaluation of entropy production is the key to describing the time evolution of an out-of-equilibrium system. MNET uses this fundamental principle to derive kinetic equations for non-equilibrium systems at small scales where fluctuations are unavoidable. In this work, we have particularized this formalism to describe VCSA. The application of MNET enables the derivation of a universal kinetic framework for VCSA based on the knowledge of the free energy of capsid formation provided by the CNTVC. This approach leads to a general Fokker–Planck equation considering capsid formation as a diffusive process in the space of mesoscopic reaction coordinates. The system’s free energy dictates the form of the general kinetic equation, Equation (18), ultimately specifying the corresponding kinetic equations for different capsid architectures. This theoretical framework offers key advantages. It allows for the calculation of the size distribution of fully formed capsids, which, due to the out-of-equilibrium kinetics, may differ significantly from the energetically most stable capsid or the one obtained following the minimum energy path. It also provides a time-dependent description of the distribution of intermediates and the rate at which viral capsids form. Furthermore, this technique offers opportunities to explain interesting phenomena in the theoretical modeling of VCSA, including the formation of capsids of suboptimal size [45,100,101] and the hypothesis that CBBs may have optimal bending angles distinct from those observed in native viruses [101]. This framework could deepen our understanding of VCSA and enable innovative applications, such as antiviral strategies based on kinetic considerations or by inducing misassembly and the design of virus-like particles with efficient and controlled assembly for bio- and nanotechnological purposes. These ideas can also be extended to describe the kinetics of formation of other nanoscale structures. 

## Data Availability

No new data were created or analyzed in this study. The original contributions presented in the study are included in the article.
